# Autologous Platelet Lysate Is an Alternative to Fetal Bovine Serum for Canine Adipose-Derived Mesenchymal Stem Cell Culture and Differentiation

**DOI:** 10.3390/ani13162655

**Published:** 2023-08-17

**Authors:** Usman Rashid, Evelyn Saba, Arfan Yousaf, Waleed Ahsan Tareen, Adeel Sarfraz, Man Hee Rhee, Mansur Abdullah Sandhu

**Affiliations:** 1 Department of Clinical Studies, Faculty of Veterinary and Animal Sciences, PMAS-Arid Agriculture University, Rawalpindi 46300, Pakistan; usmanrashid@uaar.edu.pk (U.R.); arfanyousaf@uaar.edu.pk (A.Y.); 2 Department of Veterinary Biomedical Sciences, Faculty of Veterinary and Animal Sciences, PMAS-Arid Agriculture University, Rawalpindi 46300, Pakistan; evelyn.saba@uaar.edu.pk (E.S.); waleedtareen1100@gmail.com (W.A.T.); 3 Department of Anatomy and Histology, Faculty of Veterinary and Animal Sciences, The Islamia University of Bahawalpur, Bahawalpur 63100, Pakistan; adeel.sarfraz@iub.edu.pk; 4 Department of Veterinary Medicine, College of Veterinary Medicine, Kyungpook National University, Daegu 41566, Republic of Korea

**Keywords:** dogs, fetal bovine serum, platelet rich plasma, mesenchymal stem cell, adipogenesis, osteogenesis

## Abstract

**Simple Summary:**

Fetal bovine serum is extracted from cow fetuses and is the primary source of nutrition for cell growth in laboratory cell culture media. Exclusively for research purposes, the fetal bovine serum is harmless, but for transplantation research, it can cause severe immune disease in the host. Keeping this serious ethical and scientific issue in mind, we used allogeneic platelet lysate, a nutritional supplement obtained from the same animal that will be transplanted with its stem cells, for stem cell culture. Cell culture media supplemented with different combinations of fetal bovine serum or allogeneic platelet lysate were used in this study. Parameters such as cell doubling time, cell viability, stem cell expression markers, and gene expression of various markers related to the transformation of stem cells into adipocytes and bone cells. Our results showed that with the use of an autologous platelet lysate, the values of all parameters were far better when compared to fetal bovine serum. This means that autologous platelet lysate can be used as a substitute for fetal bovine serum without changing the differentiation potential of cells, thereby addressing the ethical and scientific issues of fetal bovine serum for regenerative medicine.

**Abstract:**

The use of fetal bovine serum (FBS) in regenerative medicine raises serious ethical and scientific concerns. We have cultured and differentiated the canine mesenchymal stem cells (cMSCs) in five different media combinations of autologous platelet lysate (A-PL) and FBS; consisting of 0% A-PL and 10% FBS (M-1), 2.5% A-PL and 7.5% FBS (M-2), 5% A-PL and 5% FBS (M-3), 7.5% A-PL and 2.5% FBS (M-4), and 10% A-PL and 0% FBS (M-5). The cMSCs were evaluated for their doubling time, differentiation efficiency, and expression of CD73, CD90, CD105, and PDGFRα. The mRNA expression of *NT5E*, *THY1*, *ENG*, *PPARγ*, *FABP4*, *FAS*, *SP7*, *BGLAP*, and *SPP1* was also assessed. The results indicated non-significant differences in cellular proliferation/viability; positive expression of surface markers, and PDGFRα with substantial adipo/osteogenic differentiation. The expression of adipogenic (*PPARγ*, *FABP4*, *FAS*), and osteogenic (*SP7*, *BGLAP*, *SPP1*) genes were higher (*p* < 0.05) in the M5 group. In conclusion, A-PL in cMSCs culture did not negatively affect cellular proliferation and viability but also enhanced their genetic potential for multilineage differentiation. Our results indicate that A-PL can be used as an alternative for FBS to develop potent cMSCs under good manufacturing practice protocol for regenerative medicine.

## 1. Introduction

Stem cells (SCs) are of great significance in regenerative medicine due to their unique properties of self-regeneration, long-term viability, and multilineage differentiation ability. SCs are divided into induced pluripotent stem cells (iPSCs), mesenchymal stem cells (MSCs), and embryonic stem cells (ESCs). The MSCs have gained prominent status due to their ease of isolation and minimal ethical concerns [1]. According to the International Society of Cell Therapy, cultured MSCs must be adherent to a plastic surface, carry the positive expression of CD73, CD90, and CD105, and have multilineage differentiation capability [2]. So far, MSCs have been harvested from multiple sources, such as from the umbilical cord, bone marrow, Wharton’s jelly [3,4], and adipose tissue [5]. The adipose tissue is considered to be a rich source of MSCs because of its accessibility and production of a large number of cells with a high proliferation rate [6] and differentiation capabilities [7]. As it is known, the dog is one of the oldest companion animals. Due to their similar pathophysiology, they are an excellent choice for studying various human musculoskeletal conditions [8]. Among various sources of MSCs in canines [9,10], the infrapatellar fat pad (IPFP)-derived MSCs have a higher proliferation rate, better colony-forming capacity, and trilineage differentiation than any other adipose-derived MSC (AD-MSC) source.

In mammalian cell cultures, fetal bovine serum (FBS) supplementation is known to be a gold standard because it provides various growth factors and vital nutrients to the cultures. However, collecting serum from a fetus raises major ethical issues [11]. Apart from ethical issues, its inadaptability to a complete characterization, batch-to-batch variability [12], and contamination [13] may challenge the old views on the role of FBS in cell culture. Platelet lysate (PL), one of the important sources of growth factors released when platelets rupture, has received considerable attention in recent years due to its use in cell culture studies. PL is essentially obtained from the disruption of platelets in platelet-rich plasma (PRP). A study reported that PL more efficiently expanded MSCs in vitro by increasing proliferation potential and increasing colony size by enhancing colony-forming units [14]. Moreover, PL can substitute osteogenic agents in the pre-induction of MSCs before implantation in vivo. Thus, human PL provides a new tool suitable for expanding MSCs on a clinical scale without the use of xenogeneic substances such as FBS [15,16].

However, information on the use of PRP/PL as a substitute for FBS in MSC culture is scarce in veterinary medicine. Similarly, there is no commercially available species-specific serum replacement for canine MSC (cMSC) culture [17]. Furthermore, pooled PRP/PL or FBS is not a preferable source in regenerative medicine because of the presence of viruses and prions produced in plasma and its influence on the immune response caused by the internalization of proteins during the in vitro culture of MSC [18]. Based on previous literature, this study aimed to assess the dose-dependent effects of autologous platelet lysate (A-PL) on the in vitro characterization and differentiation potential of cMSCs as an alternative to FBS to obtain cells under a good manufacturing practice protocol.

## 2. Materials and Methods

Cell culture-grade chemicals were used in this study. Low glucose Dulbecco’s Modified Eagle’s Medium (LG-DMEM) and FBS were acquired from Bio West, Nuaillé, France. Penicillin–streptomycin, Amphotericin-B, and trypsin-EDTA (0.5% and 5.3 mM *w*/*v*, respectively) were obtained from Caisson, Smithfield, UT, USA. Minimum essential medium-alpha (α-MEM) was purchased from Gibco, Carlsbad, CA, USA, and Ex-Cyte from Millipore, Billerica, MA, USA. Insulin (3.5 mg [100 IU]/mL) was obtained from Novo Nordisk, Søborg, Denmark. MTT dye, collagenase type I, and TRIZOL (TriQuick, Catalogue #R1100) reagent were obtained from Solarbio, Fengtai, China. Dimethyl sulfoxide (DMSO), formalin, Triton X–100, isobutylmethylxanthine (IBMX), Dulbecco phosphate buffer saline (DPBS^−/−^; without Ca^2+^ and Mg^2+^), and Alizarin Red S stain (ARS) were procured from Sigma-Aldrich, Taufkirchen, Germany. The antifade mounting media was obtained from Vecta Shield, St. Neots, UK. The cDNA synthesis kit (Catalogue # cDSK01-100) was purchased from Vivantis Technologies, Selangor, Malaysia. SYBR green master mix and Oil red O (ORO) were obtained from Thermo Scientific, Chino, CA, USA. T-25, and T-75 cell culture flasks, serological pipettes, 6-well, 24-well, and 48-well cell culture plates, and cell strainers were received from Corning, NY, USA.

### 2.1. Tissue Collection

Critically injured dogs (*n* = 3) aged 6–12 months were received at the outdoor clinics of the Faculty of Veterinary and Animal Sciences, PMAS-Arid Agriculture University, Rawalpindi. After a comprehensive clinical examination and approval by the hospital veterinarian, the dogs were euthanized in accordance with the AVMA guidelines for euthanasia [19]. Afterward, the infrapatellar fat pad was isolated and kept in DPBS^−/−^ supplemented with 5%penicillin–streptomycin (100 U/mL–100 µg/mL) and Amphotericin-B (250 mg/L) solution before its transportation to the stem cell laboratory. Before conducting the experiment, all procedures were approved by the Institutional Ethical Committee, PMAS-Arid Agriculture University Rawalpindi, Pakistan.

### 2.2. Cell Isolation and Culturing

After collection, the fat samples were washed with DPBS^−/−^ supplemented with 5% penicillin–streptomycin and Amphotericin-B solution, and chopped to make a slurry. The fat slurry was exposed to enzymatic digestion for 135 min at 37 °C in LG-DMEM with collagenase type I (0.1 mg/mL). The enzymatic activity was then stopped by including an equal volume of LG-DMEM with 10% FBS. After the digested fat slurry was sieved through a 100 μm cell strainer, it was centrifuged at 300× *g* for 10 min at room temperature to isolate the cells. The cell pellet left after centrifugation was resuspended in LG-DMEM (supplemented with 10% FBS, 1% penicillin-streptomycin, and amphotericin-B), planted into T-25 cell culture flasks and kept in a humidified environment at 37 °C with 5% CO_2_. Every 48 h, the medium was replaced. Once the cells reached 80% confluence, they were treated with trypsin-EDTA working solution (0.05% (*w*/*v*) and 0.53 mM, respectively) and sub-cultured to the second passage (P-2). The freshly propagated cells at P3 were used for further experimentation.

### 2.3. Preparation of Autologous Platelet Lysate 

Before the dogs were euthanized, 15 mL of peripheral blood was collected aseptically in a 3.8% (*w*/*v*) sodium citrate anticoagulant vacutainer, and a platelet count was performed using a modified Neubauer chamber. Afterward, the blood samples were centrifuged at 101× *g* for 10 min (soft spin) and the erythrocyte fraction was discarded, whereas the plasma supernatant was collected in a sterile tube prior to a second centrifugation at 402× *g* for 10 min (hard spin). Subsequently, the lower third of the platelet-rich plasma (PRP) and buffy coat-containing platelets were collected in a separate sterile tube, and platelet count was performed in PRP using the modified Neubauer chamber. The recovered platelets were activated by adding sterile 10 µL/mL of 20% CaCl_2_ and kept at 37 °C for 30 min. The formed platelet lysate was filtered through a 0.22 µm syringe filter and kept at −20 °C until further use. The quantitative detection of platelet-derived growth factor-AB (PDGF-AB) in A-PL of individual samples was assessed by using the PDGF-AB sandwich ELISA kit (MyBioSource, San Diego, CA, USA), following the manufacturer’s instructions, with absorbance measured at 450 nm using a microplate reader (BioTek 800TS, Linden Ave N Shoreline, WA, USA).

### 2.4. Experimental Design

This experiment aimed to evaluate the dose-dependent effects of FBS and A-PL on the characterization and differentiation of canine adipose stem cells (cASCs). The general media comprising LG-DMEM, 1% penicillin–streptomycin, and Amphotericin-B, was further supplemented with the five different concentrations of FBS and A-PL, as mentioned in Table 1. For differentiation purposes, the cells were provided with a differentiation medium described elsewhere.

### 2.5. Cellular Doubling Time Assay

In P-3, the cell doubling time was calculated on the 1st, 2nd, 3rd, 4th, and 5th day of cell replication. Briefly, 1 × 10^4^ cells/well were seeded in a 48-well culture plate and provided with one of five (M1–M5) general media. For cell counting, the medium was removed and the cells were washed twice with DPBS^−/−^. A working solution of trypsin-EDTA was added for 10 min to detach the cells. Then, the trypsin activity was terminated with LG-DMEM, supplemented with one of the five culture media. Reconstituted samples were processed through a modified Neubauer chamber and resuspended in 1 mL of medium. A trypan blue exemption test (>90%) was used to ascertain their viability.

### 2.6. Cellular Metabolic Assay

On the 1st, 2nd, 3rd, 4th, and 5th day of culture, cellular viability was evaluated by 3-(4,5-dimethyl thiazole-2-Yl)-2,5-diphenyltetrazolium bromide (MTT) dye. Briefly, 1 × 10^4^ cells/well were seeded in a 48-well culture plate and provided with one of five different general media (M1–M5). For the metabolic activity of the cells, 0.25 μg/mL MTT dye was added and incubated at 37 °C in a 5% CO_2_ humidified atmosphere for 180 min. In active cells, the MTT dye was reduced by the mitochondrial dehydrogenase enzyme into blue formazan crystals. The formed cell crystals were further blended with 100 μL of DMSO, and their absorbance was measured at 630 nm using a microplate reader.

### 2.7. Cell Migration/Scratch Assay

A scratch assay (also known as a wound healing assay) was performed to assess cell migration/proliferation as previously described by Liang et al. [20] with a minor modification. We used an injection needle instead of a micropipette tip to create the scratch. For this purpose, at 95% confluence, the MSC monolayer was scratched straight with the needle, and detached cells were rinsed twice with the culture media, incubated at 37 °C, and 5% CO_2_ concentration. The scratched area was photomicrographed immediately after scratching and after 24 h. The width of the scratch at each interval (T: 0 h and T1: 24 h) was measured with ImageJ software. Cell migration was calculated with the following formula [21]:

$$\frac{Distance\;at\;T0-distance\;at\;T1}{distance\;at\;T0}\times 100$$

### 2.8. Fluorescent-Activated Cell Sorting 

The presence of cell surface antigens (CD73, CD90, and CD105) and PDGFRα on MSCs cultured in five different general media (M1–M5) was evaluated. Briefly, 5 × 10^5^ cells from different culture recipes were pelleted and re-suspended in DPBS^−/−^. The primary (rabbit polyclonal) antibodies against CD73 (E-AB-10944), CD90 (E-AB-16098), CD105 (E-AB-34276) from Elab Science, Houston, TX, USA, and PDGFRα (Orb6660, Biorbyt, Cambridge, UK) were diluted in DPBS^−/−^ (1:100) and incubated at 37 °C for 15 min. In order to remove unconjugated antibodies, the cells were washed with DPBS^−/−^ and centrifuged at 500× *g* for 5 min at room temperature. Cells were incubated for 15 min with the secondary antibody (1:300; separate tubes for each antigen) conjugated with Alexa Flour-488. After incubation, the cells were washed (300× *g*) and flow cytometry was performed immediately through FACScan (BD Biosciences, San Jose, CA, USA) using CELLQuest (BD Biosciences, version 5.2) software. 

### 2.9. Canine Mesenchymal Stem Cells Bi-Linage Differentiation

At P-3, the MSCs were differentiated into adipocytes and osteocytes. For the adipogenic differentiation, 2.5 × 10^4^ cells/well were seeded in a 24-well plate and 1 of 5 (M1–M5) mediums was provided. After the cells were checked for 90% confluence, they were treated with the adipogenic induction media composed of LG-DMEM, 0.1 mM IBMX, 10-µM rosiglitazone, 0.3 mM dexamethasone, 5 μg/mL insulin, and 1% penicillin–streptomycin and amphotericin-B, and was further supplemented with one of the concentrations of FBS and A-PL, as mentioned in Table 1, for the following 48 h. Subsequently, for the next 7 days, cells were supplemented with an adipogenic maintenance medium consisting of LG-DMEM, 1% Ex-Cyte, 5 μg/mL insulin, and 1% penicillin–streptomycin and amphotericin-B with media change every 48 h. After 7 days, for the identification of cytoplasmic fat droplets, the cells were washed twice with DPBS^−/−^, fixed with 4% buffered formalin at room temperature for 30 min, and stained with ORO (6:4 in distilled water) for 30 min at room temperature, and observed under an inverted light microscope (Labomed 400, Los Angeles, CA, USA). To quantify ORO, anhydrous isopropanol was used to elute the stain, and the absorbance was measured at 490 nm and normalized by cell number.

For the osteogenic differentiation, 2.5 × 10^4^ cells/well were seeded in a 24-well plate, and one of five (M1–M5) mediums was provided. After reaching 90% confluence, the cells were supplemented with an osteogenic growth medium, comprising alphaMEM, 10 mM ß–glycerophosphate, 50 µM ascorbate-2-phosphate, 100 nM dexamethasone, 0.75 nM vitamin D_3_, 1% penicillin–streptomycin and amphotericin-B, and supplemented with different concentrations of FBS and A-PL (M1–M5), as mentioned in Table 1. After 21 days, the extracellular mineralization was assessed with ARS staining. The cells were subjected to two DPBS^−/−^ washes and fixed with 4% buffered formalin at room temperature for 30 min prior to staining. After cells were incubated in 40 mM ARS working solution for 45 min at room temperature in the dark, the dye was washed off with DPBS^-/-^ and examined with an inverted light microscope.

### 2.10. Alkaline Phosphatase Activity

The osteogenesis was determined by the alkaline phosphatase (ALP) activity, quantified using a commercially available kit (ELITech Group, Puteaux, France), according to the manufacturer’s instructions. The *p*-nitrophenyl phosphate (*p*-NPP) worked as a substrate for cellular ALP activity, and the total protein present in the cellular lysate was used for the normalization of the enzymatic activity.

### 2.11. Gene Expression Analysis

To analyze gene expression, the cells were cultured in six-well cell culture plates and treated with general, adipogenic, and/or osteogenic differentiation medium (M1–M5) as described above. To extract total RNA, the cells were scraped with a cell scraper and centrifuged (300× *g*, 5 min), pelleted, and stored at −80 °C. Total RNA was isolated using a TRIZOL reagent according to the manufacturer’s instructions. Briefly, RNA was extracted with the help of chloroform, and the extracted supernatant was washed with propanol and ethanol. After the last washing step, the extracted RNA pellets were suspended in DEPC water (RNAse- and DNAse-free water). From RNA, cDNA was synthesized using a cDNA synthesis kit. The qPCR reaction was carried out in Galaxy XP Thermal Cycler (Bioer, Zhe Jiang Sheng, ZheJiang, China) using SYBR green master mix. In the thermocycler, the initial denaturation reaction was carried out at 95 °C for 10 min, followed by denaturation at 95 °C for 15 s, primer annealing/extension at 72 °C for 20 s (Table 2), and finally at 95 °C for 15 s. All reactions were performed in duplicate, GAPDH was used as a housekeeper, and ΔΔCT values were calculated for each gene. Real-time fluorescence monitoring of qPCR amplification products is shown in Supplementary Materials Figure S1.

### 2.12. Statistical Analysis

In this manuscript, both the statistical analysis and graphs were plotted using SigmaPlot 12.0 software (Systat Software Inc., San Jose, CA, USA). Each assay was performed in duplicate. The qPCR analysis was performed in duplicate and observations of ALP activity, flow cytometry analysis, and ORO were pooled from 2 wells of 6-, 24-, or 48-well cell culture plates. Cellular doubling time and viability assays were analyzed using two-way ANOVA. All other data (ORO quantification, ALP activity, and qPCR analysis) were analyzed by one-way ANOVA, where the interaction was maintained in the media × individual determinations. The Holm–Sidak posthoc test was used to determine the significance between the different groups. In the manuscript, all datasets are presented as means with standard error of the mean (SEM), where *p* < 0.05 indicates statistical significance.

## 3. Results

### 3.1. Isolation and Expansion of Canine Mesenchymal Stem Cells

**Figure 1:**
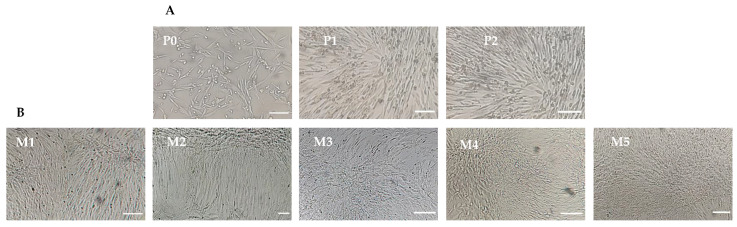
Representative pictures of the infrapatellar fat pad (IPFP)-derived cMSCs at Passage-0 (P-0), Passage-1 (P-1), and Passage-2 (P-2) (**A**) IPFP derived cMSCs from Passage-3 are grown in five types of media (**B**) 20× magnification. M1 (FBS = 10%, A-PL = 0%). M2 (FBS = 7.5%, A-PL = 2.5%). M3 (FBS = 5%, A-PL = 5%). M4 (FBS = 2.5%, A-PL = 7.5%). M5 (FBS = 0%, A-PL = 10%). Scale bar = 20 µm.

IPFP-MSCs were cultured and expanded in a complete LG-DMEM supplemented with 10% FBS until passage-2 (P-2) as shown in Figure 1A. At Passage-3 (P-3), the IPFP-MSCs were provided with five different concentrations of FBS and A-PL. Nucleated cells adhered to the surface of the culture flasks and expanded in the form of colonies, showing a typical appearance of fibroblasts (Figure 1B, M1–M5).

### 3.2. Platelets Count and Platelet-Derived Growth Factor-AB Analysis

The platelet count was performed in whole blood and PRP. The platelet count in whole blood was 47.8 × 10^4^ cells/mL, but after a hard spin, the count increased to 168 × 10^4^ cells/mL, which is about 3.51 times that of the whole blood. After the rupturing of platelets, we analyzed the presence of PDGF-AB in A-PL. The PDGF-AB concentrations obtained in the three A-PL samples were 68.32 ng/mL, 62.05 ng/mL, and 109.17 ng/mL, respectively, with an average value of 79.85 ng/mL.

### 3.3. Cellular Doubling Time

**Figure 2:**
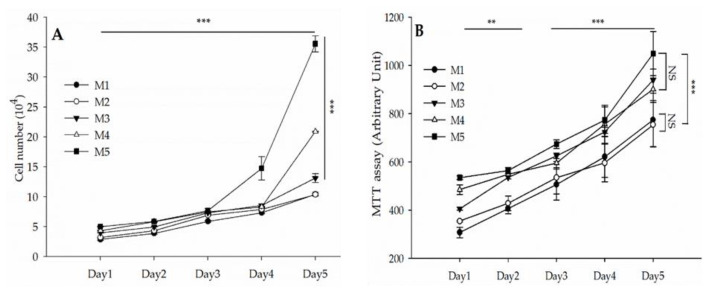
Cell doubling time and metabolism of cMSCs in different media groups; (**A**) cell doubling time at P-3, on the first, second, third, fourth, and fifth days of culture. (**B**) Cell metabolism at P-3 on the first, second, third, fourth, and fifth days of culture. Data are presented in mean ± SEM. *** *p* < 0.001 and ** *p* < 0.003. M1 (FBS = 10%, A-PL = 0%). M2 (FBS = 7.5%, A-PL = 2.5%). M3 (FBS = 5%, A-PL = 5%). M4 (FBS = 2.5%, A-PL = 7.5%). M5 (FBS = 0%, A-PL = 10%).

The cMSCs were cultured in different media to evaluate the effects of FBS and A-PL (M1–M5) on growth kinetics. The population doubling time was calculated on the first, second, third, fourth, and fifth days of culture, as shown in Figure 2A. Our results showed that the cell growth of all the media (M1–M5) groups remained non-significant on the first, second, and third days of culture. However, on the fourth day of culture, compared with the other four groups, the cell growth of the M5 medium group was significantly increased (*p* < 0.001). Similarly, compared with the M1 and M2 medium groups, cMSCs cultured in the M3 to M5 medium groups showed a significant increase in cell growth (*p* < 0.001) on the fifth day. Moreover, the media group × day interaction showed a significant (*p* < 0.001) difference between days and groups.

### 3.4. Cell Metabolism

The MTT assay was performed to evaluate the effect of different concentrations of FBS and A-PL on the metabolism of cMSCs on the first, second, third, fourth, and fifth days of culture as shown in Figure 2B. The cellular metabolism remained non-significant (*p* > 0.05) among M3 to M5 and between M1 and M2 media groups. Overall, cMSCs cultured in M3 to M5 showed significantly (*p* < 0.001) higher cellular metabolic activity than those cultured in the M1 and M2 media groups. On the other hand, the cellular metabolism increased significantly with increasing days in the culture medium.

### 3.5. Cell Migration/Scratch Assay

**Figure 3:**
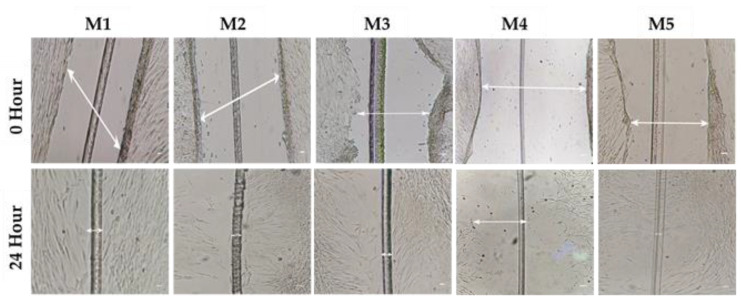
Photomicrographs of -initial defects (scratches) at 0 h using a sterile 28-gauge needle compared to 24 h in different media groups (M1–M5); 20× magnification. M1 (FBS = 10%, A-PL = 0%). M2 (FBS = 7.5%, A-PL = 2.5%). M3 (FBS = 5%, A-PL = 5%). M4 (FBS = 2.5%, A-PL = 7.5%). M5 (FBS = 0%, A-PL = 10%). The white arrow represents a gap between the scratched edges.

The wound healing assay analyzed the proliferative capacity of AD-MSCs supplemented with different concentrations of FBS and A-PL (M1–M5). Gaps were measured at 0 h and 24 h after wound induction (Figure 3). 

**Figure 4:**
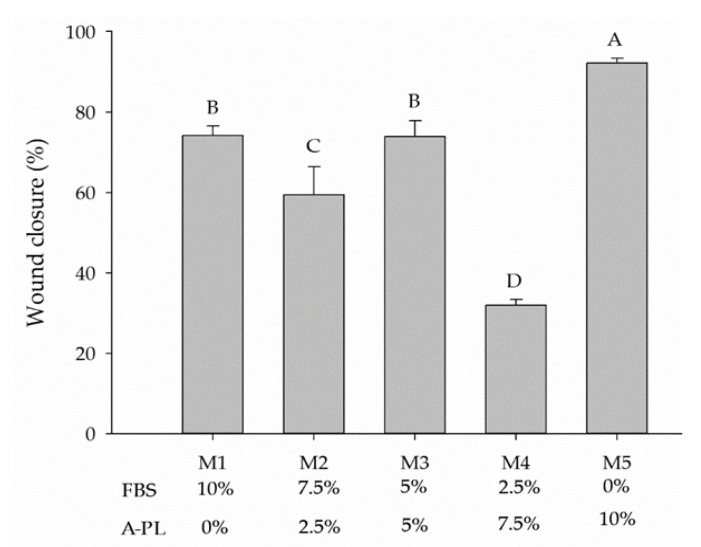
Graphical comparison of wound occlusion rates of canine infrapatellar fat pad mesenchymal stem cells cultured for 24 h in media supplemented with different concentrations of fetal bovine serum (FBS) and autologous platelet lysate (A-PL). Superscript ^A–D^ designates the significant difference (*p* < 0.05).

At 0 h, no significant difference was observed in scratch width. However, after 24 h, AD-MSCs cultured under the M5 media group (A-PL 10%) showed a significantly (*p* < 0.001) higher proliferation rate compared to all other four media groups. The monolayer of the AD-MSCs in the M5 group completely covered the initial width of the scratch (Figure 4). On the other hand, AD-MSCs cultured in M4 media groups had the slowest proliferative rate as compared to other media groups. While AD-MSCs in M1 and M3 media groups showed, non-significantly (*p* > 0.05), the same proliferative rate.

### 3.6. Immunophenotypic Analysis

**Figure 5:**
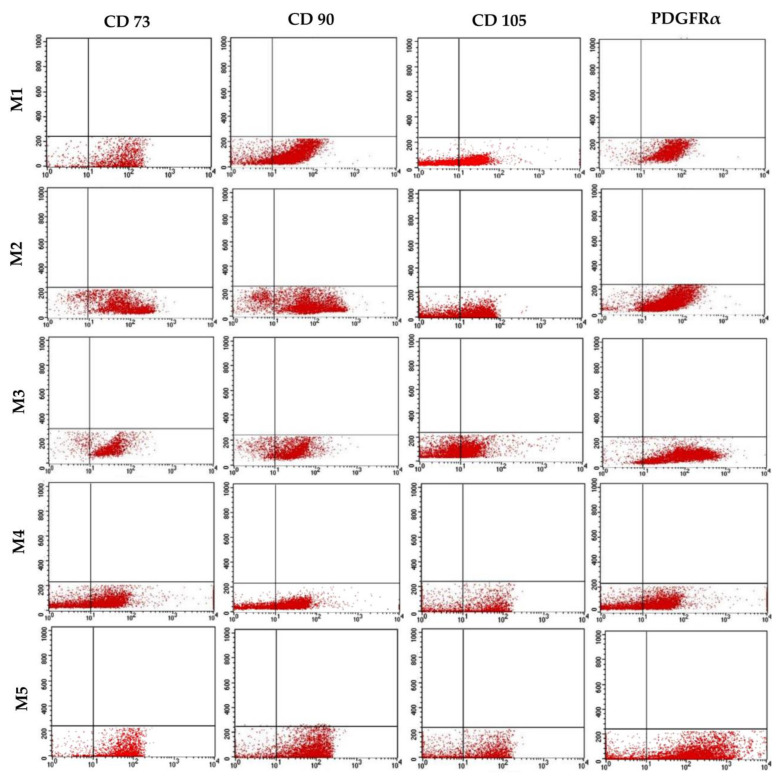
Flow cytometric expression of CD73, CD90, CD105, and PDGFRα in undifferentiated cMSCs grown in different media supplemented with fetal bovine serum and autologous platelet lysate.

The expression of stem cell markers (CD73, CD90, and CD105) and PDGFRα were evaluated using flow cytometric analysis (Figure 5). The mean (*n* = 3) expression percentages of CD73, CD90, CD105, and PDGFRα in the M1 group were 88.90%, 83.01%, 58.39%, and 95.24% respectively, whereas the expression percentages in the M2 group were 88.40%, 82.74%, 55.37%, and 92.17%, respectively. In the M3 group, the expression percentages were 88.79% (CD73), 84.33% (CD90), 56.10% (CD105), and 96% (PDGFRα). The expression percentages of CD73, CD90, CD105, and PDGFRα in the M4 and M5 groups were 81.45%, 78.21%, 48.4%, 87.99% and 87.79%, 85.18%, 55.59%, and 94.35%, respectively. 

### 3.7. Mesenchymal Stem Cells Bi-Linage Differentiation Efficiency

**Figure 6:**
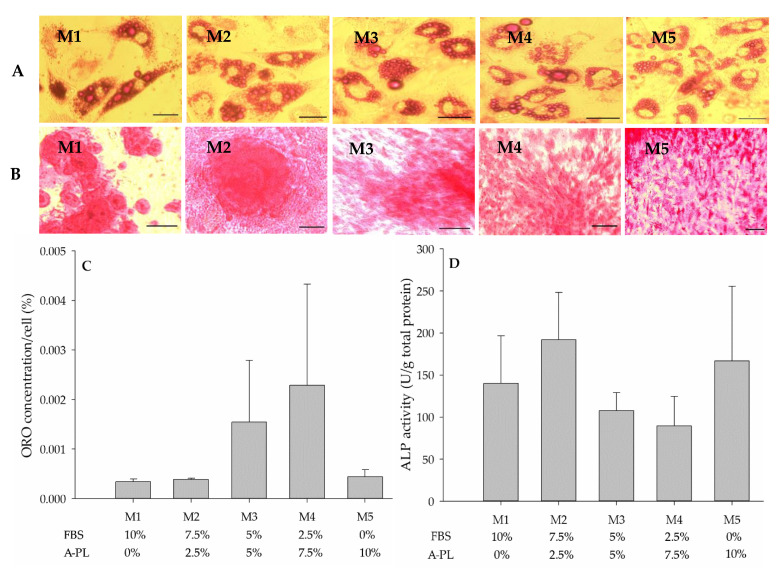
(**A**) Oil Red O (ORO) staining of adipocytes (20× magnification). Canine mesenchymal stem cells were differentiated into adipocytes in different growth media supplemented with fetal bovine serum (FBS) and autologous platelet lysate (A-PL). After 7 days of adipogenesis, the intra-cytoplasmic fat droplets stained positive with ORO. (**B**) After 21 days of culture in different osteogenic media (M1 to M5), cMSCs were stained with Alizarin Red-S (Scale bar = 20 µm); magnification 20× (**C**) ORO quantification: Anhydrous isopropanol was used to elute ORO staining from differentiated adipocytes grown in different media supplemented with FBS and A-PL. (**D**) Alkaline phosphatase (ALP) activity of differentiated osteocytes grown in different media sets supplemented with different concentrations of FBS and A-PL. Data were normalized to units/gram protein and expressed as mean ± SEM.

After 7 days of culturing in adipogenic media, cells were stained with ORO and observed under an inverted light microscope (40× magnification) for the identification of intracytoplasmic fat droplets (Figure 6A). All five media groups (M1–M5) showed insignificant (*p* = 0.631) differences in the level of adipogenesis efficiency, as shown in Figure 6C. IPFP-MSCs were cultured in different osteogenic mediums (M1–M5) for 21 days and stained with ARS to identify the deposition of hydroxyapatite minerals (Figure 6B). As an early marker of osteogenesis, the ALP activity in all five media groups remained insignificant (*p* = 0.704), as in Figure 6D.

### 3.8. Gene Expression Analysis

**Figure 7:**
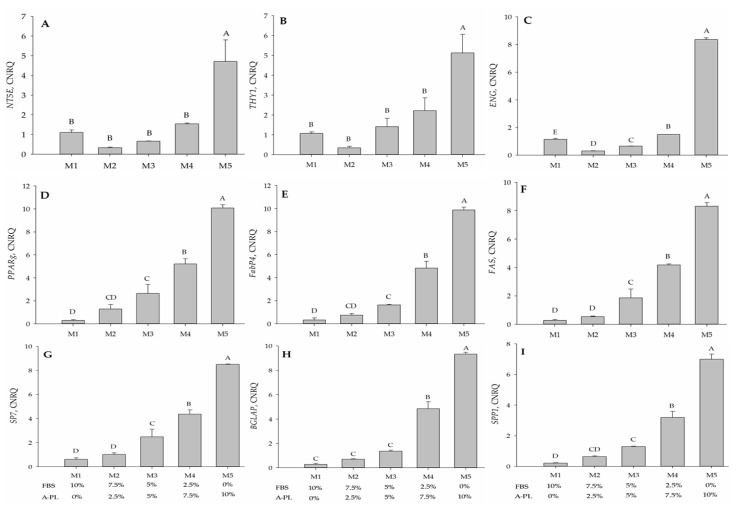
Expression of *NT5E* (**A**), *THY1* (**B**), and *ENG* (**C**) genes was assessed by culturing IPFP-derived MSCs in various concentrations of fetal bovine serum (FBS) and autologous plate lysate (A-PL). After 7 days of adipogenesis, the differentiated adipocytes were tested for the expression of *PPARγ* (**D**), *FABP4* (**E**), and *FAS* (**F**). After being kept in an osteogenic growth medium for 21 days, the expression of *SP7* (**G**), *BGLAP* (**H**), and *SPP1* (**I**) was tested in osteogenic differentiated cells. The data are expressed as mean ± SEM. Superscript ^A–E^ designates the significant difference (*p* < 0.05).

The undifferentiated IPFP-MSCs grown in different media (M1–M5) were tested for the gene expression of *CD73*, *CD90*, and *CD105*. The expression of *CD73* and *CD90* was significantly higher (*p* < 0.01) in the 10% A-PL supplemented group (M5) compared with the remaining four media (M1 to M4) groups, whereas the expression was not significantly different (*p* > 0.05) between the other four groups. Compared to the M1 (FBS 10%) group, the expression of *CD73* and *CD90* in M5 (A-PL 10%) was significantly higher (*p* < 0.003; Figure 7A,B). However, when compared with *CD73* and *CD90*, the expression of *CD105* in all five media groups was significantly different (*p* ≤ 0.01) regardless of the concentration of FBS and A-PL (Figure 7C). After 7 days of adipogenesis, the differentiated cMSCs were tested for the expression of *PPARγ*, *FABP4*, and *FAS* genes. Significantly higher (*p* < 0.01) expressions of *PPARγ* and *FABP4* were observed in groups M5 and M4 groups. The expression of *PPARγ* and *FABP4* in the M2 group was not significantly different (*p* ≥ 0.05) from that of the M1 and M3 groups. However, compared with the M1 group, the expression of the M3 group was significantly higher (*p* ≤ 0.05) (Figure 7D,E). With the gradual increase in the A-PL concentration in the medium, the expression analysis of *FAS* was enhanced significantly (*p* < 0.01) from the M3 to M5 medium group. Nonetheless, its expression remained insignificantly different (*p* = 0.556) between the M1 and M2 media groups (Figure 7F).

In addition to adipogenic genes, cMSCs cultured in osteogenic media for 21 days were tested for the expression of *SP7* (*osterix*), *BGLAP* (*osteocalcin*), and *SPP1* (*osteopontin*) genes. Between the M3 and M5 media groups, the expression of *SP7* increased significantly (*p* < 0.01), whereas a non-significantly different expression (*p* = 0.397) was observed between the media groups M2 and M1 (Figure 7G). The expression of *osteocalcin* in the M5 group was significantly higher (*p* < 0.01) than that in the M4 group, whereas the expressions of both groups M4 and M5 were significantly (*p* < 0.01) higher than the other three groups M1 to M3. The expressions of *osteocalcin* observed between the M1, M2, and M3 groups were not significantly different (*p* ≥ 0.059) (Figure 7H). In relation to A-PL concentration, a significant increase (*p* < 0.01) in the expression of *osteopontin* (Figure 7I) was observed from the M3 to M5 media groups. Although the expression of *osteopontin* in the M2 group was insignificantly different from that in the M1 (*p* = 0.222) and M3 (*p* = 0.148) media groups, its expression was significantly (*p* = 0.025) higher in the M3 than in the M1 group.

## 4. Discussion

The regenerative capability of MSCs is of great significance in regenerative medicine. FBS is a known source of growth factors for in vitro stem cell propagation and replication. However, as a xenobiotic source, the use of FBS is associated with serious scientific and ethical concerns. Considering the aforesaid concerns and the unavailability of species-specific serum supplements for cell culture, this study was designed to investigate the dose-dependent effects of A-PL alone and in combination with FBS for in vitro proliferation and differentiation of cMSCs. The cells obtained after enzymatic digestion and culture in different concentrations of A-PL and FBS exhibited typical fibroblastic morphology. The proliferation and metabolic rate of cMSCs cultured in M3 to M5 media groups showed higher proliferation as compared to those cultured in M1 and M2 media groups. The in vitro scratch test of MSCs mimics the pattern of cell migration and their proliferation in vivo. This assay can also be used to study the wound-healing process and the migration of cells to the wound site [22]. In this study, AD-MSCs cultured in the M5 medium group showed the greatest proliferative ability and filled the scratched area within 24 h. The proliferation rate of MSCs in the M1 and M3 medium groups was slightly lower than that in the M5 medium group. The AD-MSCs are known to exhibit paracrine activity and attract surrounding cells by enhancing their proliferation and differentiation capacity [23]. Our findings suggest that A-PL has a positive effect on the proliferation of MSCs. As PDGF is an important mitogen for MSCs, it plays a crucial role in wound healing and promotes fibro-proliferative and angiogenic processes [24]. The results of the scratch test also supported the results of the cell metabolism and doubling time tests, where AD-MSCs showed the greatest cell replication and metabolism in the M5 group. The similar proliferation rate in the group without FBS supplementation may be due to platelet activation leading to the degranulation of α-granules to release PDGF, TGF–ß, VEGF, IGF, and EGF [25]. PDGF is a well-known morphogen that plays an active role in wound healing by enhancing mitotic activity and thereby promoting cell proliferation [26]. PDGF and FGF-2 have the ability to initiate cellular DNA replication in the G0 or early G1 phases of the cell cycle [27]. In humans, AD-MSCs showed enhanced proliferative capacity in the presence of activated PRP [28], further supporting our findings. The cMSCs used in this study showed positive expression of CD73, CD90, CD105, and PDGFRα. Our findings are consistent with other studies using canine [9,29], bovine [30], and human [31] samples. It is known that CD73 (ecto-5′-nucleotidase) is a cell adhesion molecule [32], whereas CD90 contributes to cell migration and cell-to-cell/-matrix interactions [33]. Flow cytometry results showed that the expression of CD73 and CD90 was non-significantly different between all five treatment groups, while Sandhu et al. [30] reported a decrease in the expression of cell adhesion molecules in an FBS-free medium. Moreover, in our study, the mRNA expression of CD73 and CD90 was significantly higher (*p* < 0.05) in the M5 group, indicating that the presence of A-PL enhanced the expression of cell adhesion molecules and enabled them to proliferate as with FBS. Compared with CD73 and CD90, the expression of CD105 (endoglin) in flow cytometry was relatively low. However, in the qPCR analysis, there were significant differences in CD105 expression between all five groups (*p* < 0.05). Requicha et al. [34] pointed out that in dogs, the expression of CD105 depends on the passage numbers and tissue collection site. Our results for the expression of CD 105 are also consistent with one of our previously reported studies [35] and another study by Krešić et al. showed that the CD 105 expression in canine adipose-derived MSCs decreased as the number of cell passages increased [36]. In MSCs, reduced CD105 expression leads to the differentiation of adipocytes and osteocytes [37]. PDGFR*α* is one of two PDGF ligands (*α* and *ß*) and is known to be a promising emerging marker of MSCs [38]. The differential expression of PDGFRα in our study was insignificant between all five groups, indicating its important role in cell proliferation [26]. These findings suggested that the use of either A-PL or FBS did not affect the expression of PDGFR*α* and cellular proliferation. Irrespective of A-PL and FBS concentrations, IPFP-derived MSCs were successfully differentiated into adipocytes in the presence of rosiglitazone, IBMX, and insulin. This was further confirmed by the gene expression analysis of *PPARγ*, *FABP4*, and *FAS*. Our results showed that the expression of all three genes in the M5 group was significantly higher (*p* < 0.001) than that of other groups. The use of IBMX in combination with dexamethasone promotes *PPARγ*-induced adipogenesis [39]. Rosiglitazone is also a *PPARγ* agonist, which can accelerate the sensitivity of cells to insulin, thereby helping to stimulate the transport of glucose/cholesterol/fatty acid in developing adipocytes through the *FABP4* protein and *FAS* stimulation. Hence, *PPARγ* is considered an early marker of adipogenesis. The dose-dependent use of dexamethasone stimulates the differentiation of cells into adipogenic and osteogenic lineages [39]. Insulin is an important factor in adipogenesis, and the dose-dependent upregulation of adipogenic genes indicates the agonistic effect of insulin on platelet-rich plasma [40]. Among various growth factors present in A-PL, TGF plays an important role in adipogenesis and osteogenesis [41]. This supports the findings of our study, where the use of A-PL resulted in a higher expression of adipogenesis genes. The cMSCs cultured in different concentrations of FBS and A-PL demonstrated successful osteogenic differentiation and showed positive ARS staining of hydroxyapatite deposits. ALP is an early marker of osteogenesis [42], and our results showed non-significant differences in ALP activity between all treatments. Furthermore, the qPCR results showed that A-PL gradually increased the expression of *SP7*, *BGLAP*, and *SPP1* genes in a dose-dependent manner. The A-PL dose-dependent cell proliferation and osteogenic differentiation with a possible release of growth factors have previously been reported [43]. During the early stage of bone healing, platelets continuously release PDGF, which plays an important role in revascularization, bone healing, and remodeling [44,45]. Previous studies have reported various concentrations of A-PL ranging from 2.5% to 10% [43] of the media for a desired proliferation and differentiation of cMSCs. However, our study demonstrated much better proliferation and differentiation capabilities via the use of 10% A-PL. This may be due to the use of autologous A-PL rather than PRP or allogeneic A-PL. Our study with 10% A-PL yielded satisfactory results, but there were conflicting reports that 10% PL failed to isolate canine MSCs [46]. However, in our study, cells were isolated and propagated in FBS until P2, and in experiments at P3, cells were maintained and propagated in supplemented A-PL.

Interestingly, Even et al. [47] mentioned the comparative results of equine serum (ES), A-PL, pooled PL, and FBS on the immunomodulatory properties of BM-MSCs. According to this study, ES enhanced the immunostimulatory properties of BM-MSCs, while A-PL increased the levels of platelet-derived and inherent growth factors in BM-MSCs [47]. This study clearly supports our findings, as PL contains a wide variety of growth factors that not only promote MSC growth but also act as chemoattractants for in vivo stem cell growth.

## 5. Conclusions

In conclusion, our study recommends the use of A-PL in the cultivation of cMSCs as it provides stable biological properties and maintains cell proliferation and viability as favorably as FBS. The use of A-PL is an excellent source of growth factors, and it can be effectively used in cell culture and transplantation studies without ethical apprehensions. Nonetheless, the use of A-PL in cell cultures requires more research, especially in assessing the differences between A-PL and allogeneic/pooled A-PL in canine cell cultures.

## Figures and Tables

**Table 1:** Different concentrations of autologous platelet lysate and fetal bovine serum were used in general/differential media for canine mesenchymal stem cell growth.

Media	^1^ FBS%	^1^ A-PL%
M1	10	0
M2	7.5	2.5
M3	5	5
M4	2.5	7.5
M5	0	10

^1^ FBS = Fetal Bovine Serum; A-PL = Autologous Platelet Lysate.

**Table 2:** The primer sequences for the given cDNA from canine mesenchymal stem cells in differentiated adipocytes and osteocytes.

Gene	Sense 5′-3′	Antisense 3′-5′	Amplicon Size (BP)	Annealing Temp. (°C)
*NT5E*	TTTGGGGAAACCTTTGACC	AGAGGCTCGTAACTGGGTACTC	116	54.1
*THY1*	CGGCTTCACCACCAAGGACG	TCTGGGCCAGCAGGCTTATG	140	57.6
*ENG*	CCTCAGTGCAAAGAAGAAT	CTTGGAAGATCAGTTTGGGG	89	51.4
*FAS*	GGCTGGAGCCGGCTACTGCC	ATTCAGGATGGTAGCGTACA	94	56.7
*FABP4*	CACCATTAAATCAGAAAGCACC	CCAGGACACCTCCATCTAAG	128	50.9
*PPARγ*	TAAAGAGCCTGAGAAAGCC	GCTTCACATTCAGCAAACC	156	51.8
*SP7*	TGCTTGAGGAGGAAGCTCAC	TTTGGGGGCTGAAAGGTCAC	161	58.3
*BGLAP*	TGCAACCTTCGTGTCCAAG	TGGAAGCCAATGTGGTCAG	171	59
*SPP1*	TGATTTTCCCACTGACATTCC	TCCATACTCGCACTTTTCAC	195	53
*GAPDH*	AAGAAGGTAGTGAAGCAGG	GCGTCGAAGGTGGAAGAGTGGG	212	54.1

*NT5E* (CD73); *THY1* (CD90); *ENG* (CD105); *FAS* (Fatty acid synthase); *FABP4* (Fatty acid binding protein); *PPARγ* (Peroxisome proliferator-activated receptor-gamma); *SP7* (Osterix); *BGLAP* (Osteocalcin); *SPP1* (Osteopontin); *GAPDH* (Glyceraldehyde 3-phosphate dehydrogenase).

## Data Availability

The datasets analyzed during the current study is already detailed/provided in the manuscript.

## Supplementary Materials

The following supporting information can be downloaded at: https://www.mdpi.com/article/10.3390/ani13162655/s1, Figure S1. Melting curves were acquired by multiplex fluorescence melting curve analysis. The first derivative curve is plotted as 𝑑𝐹/𝑑𝑇 (*y*-axis) verses temperature (oC; x-axis), depicting amplification of the desired product only. In case of dimer formation, a double peak appears in the temperature range of 75 °C to 80 °C.
