# Supplementary material for: Autologous Platelet Lysate Is an Alternative to Fetal Bovine Serum for Canine Adipose-Derived Mesenchymal Stem Cell Culture and Differentiation
Source: Animals (Basel). 2023 Aug 17;13(16):2655. doi: 10.3390/ani13162655 (PMC10451755; doi:10.3390/ani13162655)
Supplement: Supplementary file 1 [file animals-13-02655-s001.zip › animals-2517089-supplementary.pdf]

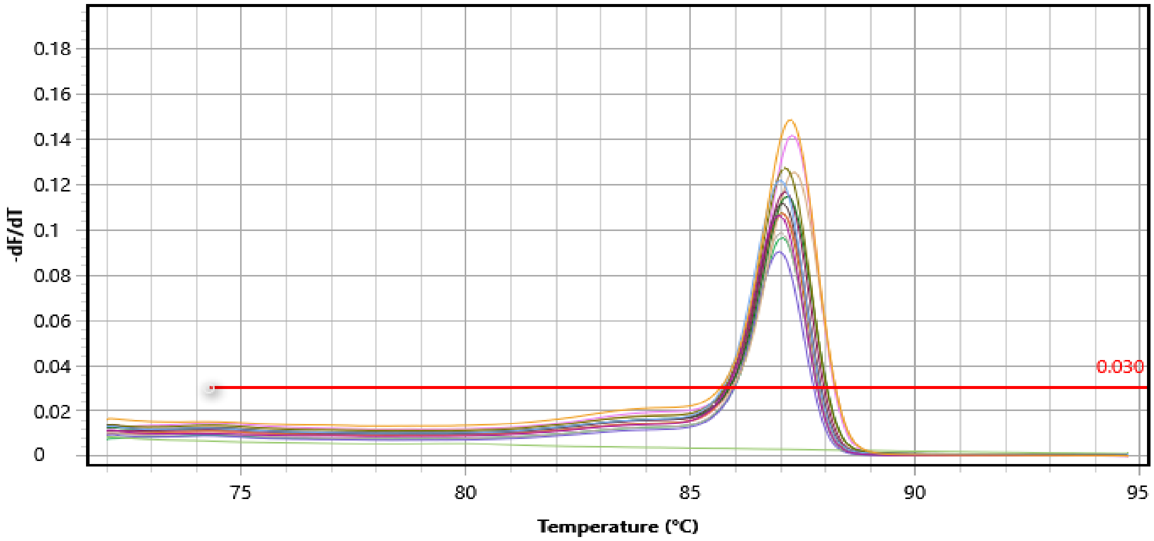

Figure S1. Melting curves were acquired by multiplex fluorescence melting curve analysis. The first derivative curve is plotted as dF/dT (y-axis) verses temperature (°C; x-axis), depicting amplification of the desired product only. In case of dimer formation, a double peak appears in the temperature range of 75 ˚C to 80 ˚C.
